# Variation and Evolution of the Whole Chloroplast Genomes of *Fragaria* spp. (Rosaceae)

**DOI:** 10.3389/fpls.2021.754209

**Published:** 2021-10-14

**Authors:** Chenxin Li, Chaonan Cai, Yutian Tao, Zhongshuai Sun, Ming Jiang, Luxi Chen, Junmin Li

**Affiliations:** ^1^College of Life Sciences and Medicine, Zhejiang Sci-Tech University, Hangzhou, China; ^2^Zhejiang Provincial Key Laboratory of Plant Evolutionary Ecology and Conservation, Taizhou University, Taizhou, China; ^3^School of Advanced Study, Taizhou University, Taizhou, China

**Keywords:** *Fragaria*, chloroplast genome, comparative analysis, wild species, phylogenetic

## Abstract

Species identification is vital for protecting species diversity and selecting high-quality germplasm resources. Wild *Fragaria* spp. comprise rich and excellent germplasm resources; however, the variation and evolution of the whole chloroplast (cp) genomes in the genus *Fragaria* have been ignored. In the present study, 27 complete chloroplast genomes of 11 wild *Fragaria* species were sequenced using the Illumina platform. Then, the variation among complete cp genomes of *Fragaria* was analyzed, and phylogenetic relationships were reconstructed from those genome sequences. There was an overall high similarity of sequences, with some divergence. According to analysis with mVISTA, non-coding regions were more variable than coding regions. Inverted repeats (IRs) were observed to contract or expand to different degrees, which resulted in different sizes of cp genomes. Additionally, five variable loci, *trnS*-*trnG*, *trnR*-*atpA*, *trnC*-*petN*, *rbcL*-*accD*, and *psbE*-*petL*, were identified that could be used to develop DNA barcoding for identification of *Fragaria* species. Phylogenetic analyses based on the whole cp genomes supported clustering all species into two groups (A and B). Group A species were mainly distributed in western China, while group B contained several species from Europe and Americas. These results support allopolyploid origins of the octoploid species *F. chiloensis* and *F. virginiana* and the tetraploid species *F. moupinensis* and *F. tibetica*. The complete cp genomes of these *Fragaria* spp. provide valuable information for selecting high-quality *Fragaria* germplasm resources in the future.

## Introduction

The genus *Fragaria* Linnaeus belongs to the family Rosaceae and is comprised of 25 species, including 13 diploids (2n), five tetraploids (4n), one pentaploid (5n), one hexaploid (6n), three octoploids (8n), and two decaploids (10n; Staudt, 1962, 1989, 2009; Davis et al., 2010; Hummer, 2012; Lei et al., 2017). Most *Fragaria* species are wild, except for *F. × ananassa*, which is a cultivated species and an economically important crop (Staudt, 1962; Potter et al., 2000; Cheng et al., 2017). China has been recognized as an important distribution center of wild strawberry resources in the world, as it has 14 wild *Fragaria* species including nine diploid species and five tetraploid species (Staudt, 1989, 2006; Lei et al., 2017). Compared with cultivated species, wild species have several advantages, including stronger resistance (Diamanti et al., 2012; Guo et al., 2018), unique fruit aromas (Urrutia et al., 2017), and richness in nutrients (Capocasa et al., 2008a,b; Tulipani et al., 2008). Therefore, elucidating the relationships among *Fragaria* spp. is vital to developing high-quality strawberry varieties.

As well as being an important plastid functioning in photosynthesis and carbon fixation (Neuhaus and Emes, 2000), the chloroplast (cp) contains a genome that can provide valuable information for taxonomic and phylogenic purposes, with the key advantages of its relatively small size, more conservative structure, and low nucleotide substitution rate (Palmer, 1985; Wolfe et al., 1987; Jansen et al., 2005; Wei et al., 2006; Wicke et al., 2011; Terakami et al., 2012; Liu et al., 2019). Furthermore, the cp genome is haploid and uniparentally inherited, so it is helpful for tracing source populations and conducting phylogenetic studies to resolve complex evolutionary relationships (Jansen et al., 2007; Parks et al., 2009; Ruhsam et al., 2015). To date, phylogenetic relationships based on complete cp genomes of many angiosperms have been fully studied in many genera (Hu et al., 2016; Zhang et al., 2016; Zhao et al., 2018). Njuguna et al. (2013) systematically studied the phylogenetic relationships of *Fragaria* based on cp genomes, but there were only 10 sequences assembled from total genomic data, and the coverage of PCR amplified sequences ranged from 60 to 90%, which may mean the assembled chloroplast sequences were incomplete. To the best of our knowledge, there is only one study that has focused on the molecular phylogenetic analysis of *Fragaria* genus based on whole complete chloroplast genome sequences (Sun et al., 2021), which revealed that 20 *Fragaria* species were clustered into northern group (eight species), southern group (11 species), and an oldest extant species (one species) based on whole cp genomes. However, this previous study focused on molecular clock analysis and ignored the variation and evolution of whole cp genomes of *Fragaria*, including, for example, the contraction and expansion of inverted repeats (IRs) regions (Plunkett and Downie, 2000; Kode et al., 2005; Ma et al., 2014; Lei et al., 2016).

In addition, although some *Fragaria* species can be identified by their morphological characteristics, some species that have similar morphological structures, such as *F. mandshurica* and *F. orientalis* (Staudt, 2003; Lei et al., 2017), are likely to be inaccurately identified if morphological indexes are not collected (Yang et al., 2020). Furthermore, Yang et al. (2020) found that most wild *Fragaria* in Yunnan were difficult to accurately identify without flowers and fruits of collected species. In total, owing to the stage of species development and subjective factors (such as differences in personal knowledge and experience), traditional morphology classification is often inconsistent and unreliable, which can also affect the results of species identification (Yang et al., 2020). Over the years, many molecular analyses have provided insights into the species taxonomy and identification. Currently, DNA barcoding such as *rbcL*, *matK*, *psbA*-*trnH* and ITS sequences (Potter et al., 2007; Fazekas et al., 2008; Chen et al., 2013; Xin et al., 2013; Tegally et al., 2019; Phi et al., 2020; Islam et al., 2021) has been used for species identification. In *Fragaria*, the study of DNA barcoding dates back to the phylogenetic analysis conducted by Potter et al. (2000) using ITS and *trnL*-*trnF* sequences, but the authors were unable to completely distinguish among species in this genus. Njuguna and Bassil (2011) analyzed *psbA*-*trnH* and ITS sequences and reported that the two DNA barcoding sequences are not suitable for species identification in *Fragaria*. Moreover, more and more studies demonstrated that the four universal barcoding sequences were problematic with low bootstrap support and inability to distinguish between species in land plants (Xin et al., 2013; Tegally et al., 2019; Phi et al., 2020; Islam et al., 2021). Therefore, it is urgent to excavate superior DNA barcoding for special land plants, including *Fragaria* species, utilizing complete cp genomes, which contain more important variation information for taxonomic and phylogenic purposes (Huang et al., 2014).

In the present study, we sequenced 27 complete cp genomes of 11 wild *Fragaria* species from different collection sites in China and downloaded the whole cp genome sequences of seven more *Fragaria* species. This research had the following objectives: (1) to describe the characteristics of *Fragaria* cp genomes; (2) to infer the phylogenetic relationships among *Fragaria* spp.; (3) to detect the variations among *Fragaria* cp genomes and infer the evolution of the whole cp genomes of *Fragaria* species; and (4) to provide candidate DNA barcodes for *Fragaria* species identification. These results provide new insights into the interspecies relationships and evolution of *Fragaria* spp. as well as basic reference material for the application of *Fragaria* germplasm resources.

## Materials and Methods

### Plant Material Collection and Genome Data Sources

Twenty-seven individuals belonging to 11 *Fragaria* species were included in the present study, as summarized in Table 1. All the sampled plants were cultured in the greenhouse facilities of Taizhou University under conditions of 70% relative humidity with temperatures of 25°C in the day and 20°C at night. The plants were identified by Professor Beifen Yang of Taizhou University. The specimens were stored in Zhejiang Provincial Key Laboratory of Plant Evolutionary Ecology and Conservation, Taizhou University, China.

**Table 1:** *Fragaria* species collection information.

Voucher	Ploidy	Locality	Latitude (N)	Longitude (E)	Altitude (m)	Genbank accession
*F. nilgerrensis*_1	2	Yunnan, China	27.01°	100.12°	3,403.50	MZ851761
*F. nilgerrensis*_2	2	Yunnan, China	25.32°	100.13°	2,159.26	MZ851762
*F. mandshurica*_JL	2	Jilin, China	42.23°	128.17°	1,047.00	MZ851758
*F. mandshurica*_HLJ	2	Heilongjiang, China	52.41°	125.14°	350.00	MZ851757
*F. corymbosa*_JL	4	Jilin, China	42.09°	128.00°	1,530.00	MZ851750
*F. corymbosa*_XZ	4	Tibet, China	29.61°	94.70°	4,082.81	MZ851751
*F. corymbosa*_GS	4	Gansu, China	35.77°	103.96°	2,833.09	MZ851749
*F. moupinensis*_XZ	4	Tibet, China	29.76°	94.73°	3,381.00	MZ851760
*F. moupinensis*_SC	4	Sichuan, China	29.21°	94.22°	3,062.85	MZ851759
*F. pentaphylla*_1	2	Qinghai, China	36.65°	101.48°	2,399.77	MZ851764
*F. pentaphylla*_2	2	Qinghai, China	36.98°	102.43°	2,327.00	MZ851765
*F. pentaphylla*_3	2	Tibet, China	28.07°	86.00°	3,261.00	MZ851766
*F. pentaphylla*_4	2	Gansu, China	35.83°	104.12°	1,966.78	MZ851767
*F. nubicola*	2	Tibet, China	28.06°	85.99°	3,353.00	MZ851763
*F. daltoniana*_1	2	Tibet, China	28.07°	86.00°	3,261.00	MZ851752
*F. daltoniana*_2	2	Tibet, China	28.07°	86.00°	3,261.00	MZ851753
*F. daltoniana*_3	2	Tibet, China	28.02°	85.98°	2,724.00	MZ851754
*F. daltoniana*_4	2	Tibet, China	28.03°	85.98°	2,956.00	MZ851755
*F. daltoniana*_5	2	Tibet, China	28.03°	85.98°	2,956.00	MZ851756
*F. viridis*	2	Segovia, Spain	41.42°	−3.76°	1,170.00	MZ851772
*F. vesca* ssp. *bracteata*	2	California, United States	38.77°	−120.45°	1,044.00	MZ851768
*F. vesca* ssp. *vesca*_1	2	Valcea, Romania	46.43°	23.76°	355.61	MZ851769
*F. vesca* ssp. *vesca_*2	2	Sichuan, China	30.04°	101.82°	3,868.00	MZ851770
*F. vesca* ssp. *vesca_*3	2	Xinjiang, China	43.87°	85.38°	1,468.00	MZ851771
*F. chinensis*_1	2	Shaanxi, China	33.27°	108.30°	1,186.95	MZ851747
*F. chinensis*_2	2	Shaanxi, China	33.27°	108.30°	1,186.95	MZ851748
*F. × ananassa*	8	Taizhou, China	28.66°	121.39°	22.69	MZ851773

The complete cp genome sequences of *F. orientalis* (NC_035501), *Fragaria chiloensis* (NC_019601), and *F. virginiana* (NC_019602) were downloaded from the National Center for Biotechnology Information (NCBI). *Fragaria nipponica* (KY769125) and *F. iinumae* (KC507759) were excluded for there is no supporting of publication reference. *Fragaria × ananassa* (KY358226) was also downloaded from NCBI to test the accuration of the sequencing and *de novo* assembly of cp genome.

Raw sequence data from three species, including *F. gracilis* (BOP214815), *F. tibetica* (BOP214818), and *F. moschata* (BOP214819; Sun et al., 2021), were downloaded from NCBI and *de novo* assembly was performed according to the following processes. The other seven species listed by Sun et al. (2021) were excluded for duplication of our own sequencing species.

### Genome Sequencing and Assembly

Fresh and clean leaves of each sampled species were collected and frozen in liquid nitrogen immediately. The samples were used to extract the total DNA by the modified CTAB method (Doyle and Doyle, 1987). Then, paired-end sequencing (Insert size: 350bp) was performed using the Illumina Novaseq 6000 platform (Illumina, San Diego, CA, United States). Raw reads were filtered to obtain high-quality clean data. Then, *de novo* assembly was performed with the GetOrganelle software package (Jin et al., 2020).

### Chloroplast Genome Annotation

Thirty cp genomes were annotated using the online GeSeq tool (Tyagi et al., 2020)^1^ with default parameters and *F. chiloensis* (NC_019601) was used as the reference to predict protein-coding genes (PCGs), transfer RNA (tRNA) genes, and ribosomal RNA (rRNA) genes. Then, the cp genome sequences were manually modified using Geneious Prime 2021.1.1 (Biomatters Ltd., Auckland, New Zealand). A circular diagram of the cp genomes was generated using the online OrganellarGenome DRAW tool (OGDRAW; Lohse et al., 2013; Bai et al., 2017).

### Comparative Genome Analyses

The boundaries of the large single-copy region (LSC), short single-copy region (SSC), and IRs of 12 newly assembled complete cp genomes of *Fragaria* species (*F. nilgerrensis*_2, *F. mandshurica*_JL, *F. corymbosa*_GS, *F. moupinensis*_SC, *F. pentaphylla*_3, *F. nubicola*, *F. daltoniana*_1, *F. viridis*, *F. vesca* ssp. *bracteata*, *F. vesca* ssp. *vesca*_1, *F. chinensis*_1, *F. × ananassa*) in this study together with six complete cp genomes of *F. gracilis* (BOP214815), *F. tibetica* (BOP214818), *F. moschata* (BOP214819), *F. orientalis* (NC_035501), *F. chiloensis* (NC_019601), and *F. virginiana* (NC_019602) from NCBI were compared using IRscope software (Ali et al., 2018). The level of divergence among the 18 sequences was visualized with Shuffle-LAGAN mode (Cheng et al., 2017; Tyagi et al., 2020) in mVISTA software (Kawabe et al., 2018; Jeon and Kim, 2019) with the default settings, and *F. gracilis* was used as a reference sequence.

### Hypervariable Site Identification

All 18 sequences were aligned using the Geneious prime 2021.1.1 plugin MAFFT v.7.450 (Katoh and Standley, 2013), and the alignment was manually adjusted. Then, we performed a sliding window analysis using DnaSP v6 software (Rozas et al., 2017; Jeon and Kim, 2019) to analyze nucleotide diversity (*π*) in order to detect hypervariable sites among *Fragaria* cp genomes. The window length was set to 600bp, and the step size was set to 200bp (Sun et al., 2021).

### Phylogenetic Analysis

Thirty-four complete cp genome sequences were used to reconstruct the phylogenetic relationships among *Fragaria* spp. based on maximum likelihood (ML) using RAxML 8.2.10 (Stamatakis, 2014), with 1,000 bootstrap replicates employed for estimating node support. *Potentilla fruticosa* (NC_036423) and *Drymocallis saviczii* (NC_050966) were downloaded from Genbank and used as outgroups (Eriksson et al., 1998, 2003; Potter et al., 2000; Feng et al., 2017; Dimeglio et al., 2014). In total, 36 cp genome sequences were aligned using a Geneious prime 2021.1.1 plugin MAFFT v.7.450 (Katoh and Standley, 2013), and the alignment was manually adjusted when necessary. Modeltest 3.7 (Liu et al., 2013) was used to select the best-fit evolutionary model of *Fragaria* cp genome sequence evolution for reconstruction of the phylogenetic relationships of *Fragaria*.

## Results

### General Chloroplast Genome Characteristics

**Figure 1:**
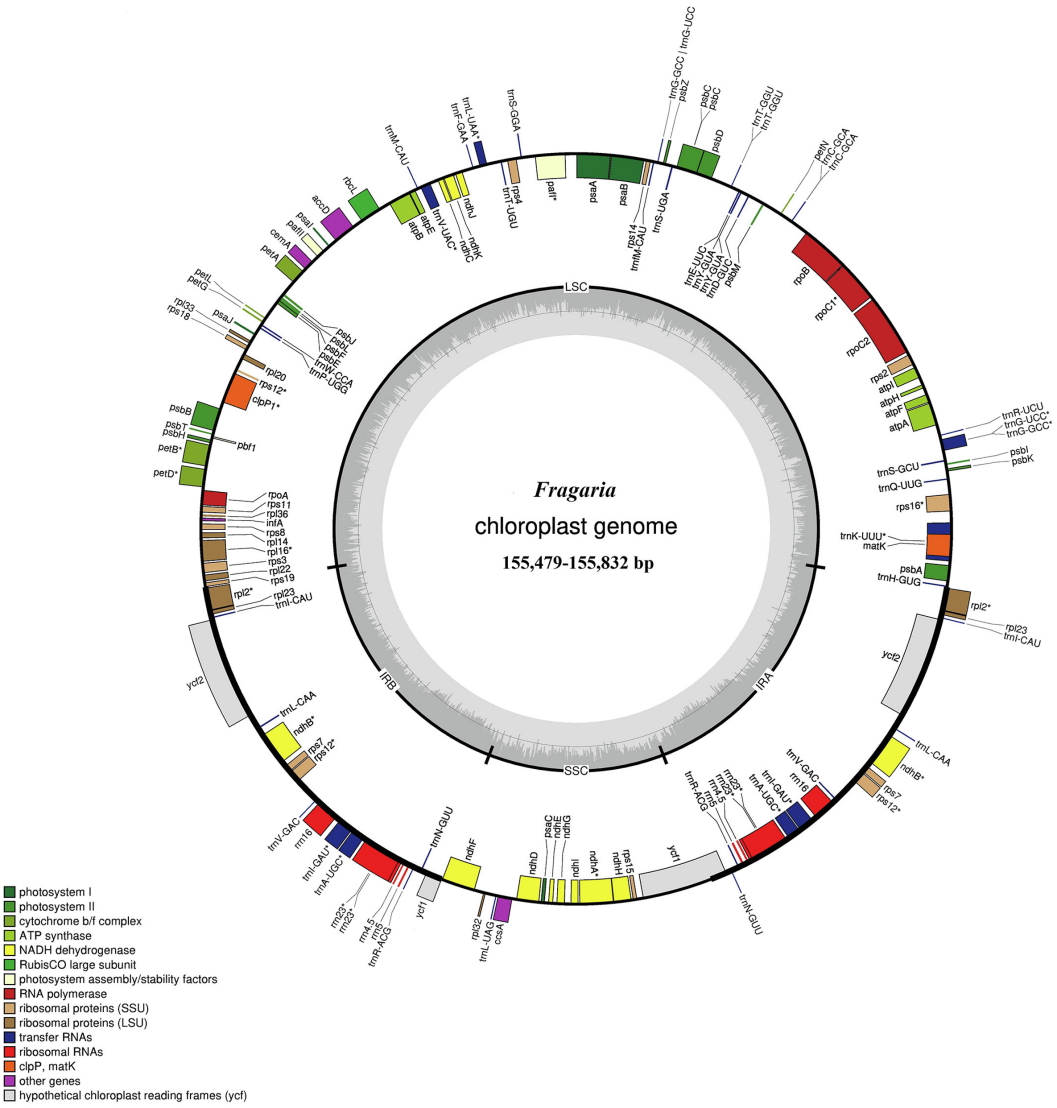
Gene map of the *Fragaria* chloroplast genome. Genes drawn inside the circle are transcribed clockwise, and those outside are transcribed counter clockwise. Color coding is used to show genes belonging to different functional groups. The inner circle indicates the range of the long single-copy region (LSC), short single-copy region (SSC), and inverted repeat regions (IRs) and also shows a GC content graph of the genome. In the GC content graph, the dark gray lines indicate GC content, while light gray lines indicate the AT content of each locus.

The total genome sequence lengths of *Fragaria* species ranged from 155,479bp (*F. viridis*) to 155,832bp (*F. daltoniana*_3). The cp genomes presented a typical quadripartite structure including a pair of IR regions with lengths of 51,872bp (*F. vesca* ssp*. bracteata*) to 51,948bp (*F. corymbosa_*JL), separated by a LSC region from 85,471bp (*F. viridis*) to 85,726bp (*F. daltoniana*_3) and a SSC region from 18,116bp (*F. viridis*) to 18,219bp (*F. moupinensis_*XZ). The GC contents ranged from 37.2 to 37.3%. Overall, the cp genome of *Fragaria* encodes a total of 130 genes, including 85 PCGs, 37 tRNA genes, and eight rRNA genes (Figure 1; Table 2). Among these genes, 15 contained a single intron (*ndhA*, *ndhB*, *petB*, *petD*, *rpl2*, *rpl16*, *rpoC1*, *rps12*, *rps16*, *trnA*-*UGC*, *trnG*-*GCC*, *trnI*-*CAU*, *trnK-UUU*, *trnL*-*UAA*, and *trnV*-*UAC*), while two harbored two introns (*ycf3* and *clpP*). The *trnK-UUU* gene had the longest intron (2,492–2,496bp), which contained the *matK* gene, whereas *trnL-UAA* was smallest (420bp; Table 3).

**Table 2:** Summary of 34 complete chloroplast genomes for *Fragaria* species.

Species	Size (bp)				Gene number				GC content (%)	References
	Total	LSC	SSC	IR	Total	PCG	tRNA	rRNA		
*F. nilgerrensis_*1	155,783	85,712	18,165	25,953	130	85	37	8	37.3	This study
*F. nilgerrensis_*2	155,675	85,602	18,147	25,963	130	85	37	8	37.2	This study
*F. mandshurica_*JL	155,559	85,504	18,161	25,947	130	85	37	8	37.2	This study
*F. mandshurica_*HLJ	155,556	85,507	18,155	25,947	130	85	37	8	37.2	This study
*F. corymbosa_*JL	155,684	85,538	18,198	25,974	130	85	37	8	37.2	This study
*F. corymbosa_*XZ	155,683	85,544	18,217	25,961	130	85	37	8	37.2	This study
*F. corymbosa_*GS	155,696	85,557	18,217	25,961	130	85	37	8	37.2	This study
*F. moupinensis_*XZ	155,630	85,487	18,219	25,962	130	85	37	8	37.2	This study
*F. moupinensis_*SC	155,626	85,489	18,215	25,961	130	85	37	8	37.2	This study
*F. pentaphylla_*1	155,626	85,510	18,192	25,962	130	85	37	8	37.2	This study
*F. pentaphylla_*2	155,640	85,524	18,192	25,962	130	85	37	8	37.2	This study
*F. pentaphylla_*3	155,628	85,511	18,193	25,962	130	85	37	8	37.2	This study
*F. pentaphylla_*4	155,666	85,549	18,193	25,962	130	85	37	8	37.2	This study
*F. nubicola*	155,608	85,512	18,174	25,961	130	85	37	8	37.2	This study
*F. daltoniana_*1	155,829	85,723	18,170	25,968	130	85	37	8	37.2	This study
*F. daltoniana_*2	155,829	85,723	18,170	25,968	130	85	37	8	37.2	This study
*F. daltoniana_*3	155,832	85,726	18,170	25,968	130	85	37	8	37.2	This study
*F. daltoniana_*4	155,827	85,721	18,170	25,968	130	85	37	8	37.2	This study
*F. daltoniana_*5	155,827	85,721	18,170	25,968	130	85	37	8	37.2	This study
*F. viridis*	155,479	85,471	18,116	25,946	130	85	37	8	37.2	This study
*F. vesca* ssp. *bracteata*	155,564	85,541	18,151	25,936	130	85	37	8	37.2	This study
*F. vesca* ssp. *vesca*_1	155,607	85,520	18,191	25,948	130	85	37	8	37.3	This study
*F. vesca* ssp. *vesca_*2	155,638	85,559	18,173	25,953	130	85	37	8	37.2	This study
*F. vesca* ssp. *vesca_*3	155,564	85,521	18,147	25,948	130	85	37	8	37.2	This study
*F. chinensis*_1	155,806	85,696	18,184	25,963	130	85	37	8	37.2	This study
*F. chinensis_*2	155,797	85,688	18,183	25,963	130	85	37	8	37.2	This study
*F. × ananassa*	155,549	85,532	18,145	25,936	130	85	37	8	37.2	This study
*F. × ananassa*	155,549	85,532	18,145	25,936	130	85	37	8	37.2	Cheng et al., 2017
*F. orientalis*	147,835	83,233	13,386	25,608	128	84	36	8	37.6	Han et al., 2018
*F. chiloensis*	155,603	85,567	18,146	25,945	130	85	37	8	37.2	Salamone et al., 2013
*F. virginiana*	155,621	85,586	18,145	25,945	130	85	37	8	37.2	Salamone et al., 2013
*F. gracilis*	155,684	85,538	18,198	25,974	130	85	37	8	37.2	Sun et al., 2021
*F. tibetica*	155,643	85,498	18,219	25,963	130	85	37	8	37.2	Sun et al., 2021
*F. moschata*	155,601	85,572	18,127	25,951	130	85	37	8	37.2	Sun et al., 2021

LSC, large single-copy region; SSC, small single-copy region; IR, inverted repeat; PCG, Protein-coding gene.

**Table 3:** Genes in the *Fragaria* chloroplast genome.

Category	Gene group	Gene name
Photosynthesis	Subunits of photosystem I	*psaA*, *pasB*, *psaC*, *psaI*, *psaJ*
	Subunits of photosystem II	*psbA*, *psbB*, *psbC*, *psbD*, *psbE*, *psbF*, *psbH*, *psbI*, *psbJ*, *psbK*, *pabL*, *psbM*, *psbN*, *psbT*, *psbZ*
	Subunits of NADH dehydrogenase	*ndhA*^a^, *ndhB*^a^^,^^c^, *ndhC*, *ndhD*, *ndhE*, *ndhF*, *ndhG*, *ndhH*, *ndhI*, *ndhJ*, *ndhK*
	Subunits of cytochrome b/f complex	*petA*, *petB*^a^, *petD*^a^, *petG*, *petL*, *petN*
	Subunits of ATP synthase	*atpA*, *atpB*,*atpE*,*atpF*, *atpH*, *atpI*
	Large subunit of rubisco	*rbcL*
Self-replication	Large subunit of ribosome	*rpl2*^a^^,^^c^, *rpl14*, *rpl16*^a^, *rpl20*, *rpl22*^a^, *rpl23*^c^, *rpl32*, *rpl33*, *rpl36*
	Small subunit of ribosome	*rps2*, *rps3*, *rps4*, *rps7*^c^, *rps8*, *rps11*, *rps12*^a^^,^^c^, *rps14*, *rps15*, *rps16*^a^, *rps18*, *rps19*
	Subunits of RNA polymerase	*rpoA*, *rpoB*, *rpoC1*^a^, *rpoC2*
	Ribosomal RNA genes	*rrn16*^c^, *rrn23*^c^, *rrn4.5*^c^, *rrn5*^c^
	Transfer RNA genes	*trnA-UGC*^a^^,^^c^, *trnC-GCA*, *trnD-GUC*, *trnE-UUC*, *trnF-GAA*, *trnG-GCC*^a^, *trnG-UCC*, *trnH-GUG*, *trnI-CAU*^c^, *trnI-GAU*^a^^,^^c^, *trnK-UUU*^a^, *trnL-CAA*^c^, *trnL-UAA*^a^, *trnL-UAG*, *trnfM-CAU*, *trnM-CAU*, *trnN-GUU*^c^, *trnP-UGG*, *trnQ-UUG*, *trnR-ACG*^c^, *trnR-UCU*, *trnS-GCU*, *trnS-GGA*, *trnS-UGA*, *trnT-GGU*, *trnT-UGU*, *trnV-GAC*^c^, *trnV-UAC*^a^, *trnW-CCA*, *trnY-GUA*
Other genes	Maturase	*matK*
	Protease	*clpP* ^b^
	Envelope membrane protein	*cemA*
	Acetyl-CoA carboxylase	*accD*
	c-type cytochrome synthesis gene	*ccsA*
Genes of unknown function	Conserved open reading frames	*ycf1*^c^, *ycf2*^c^, *ycf3*^b^, *ycf4*

a Gene with one intron.

b Gene with two introns.

c Genes with two copies.

### Comparative Analyses

**Figure 2:**
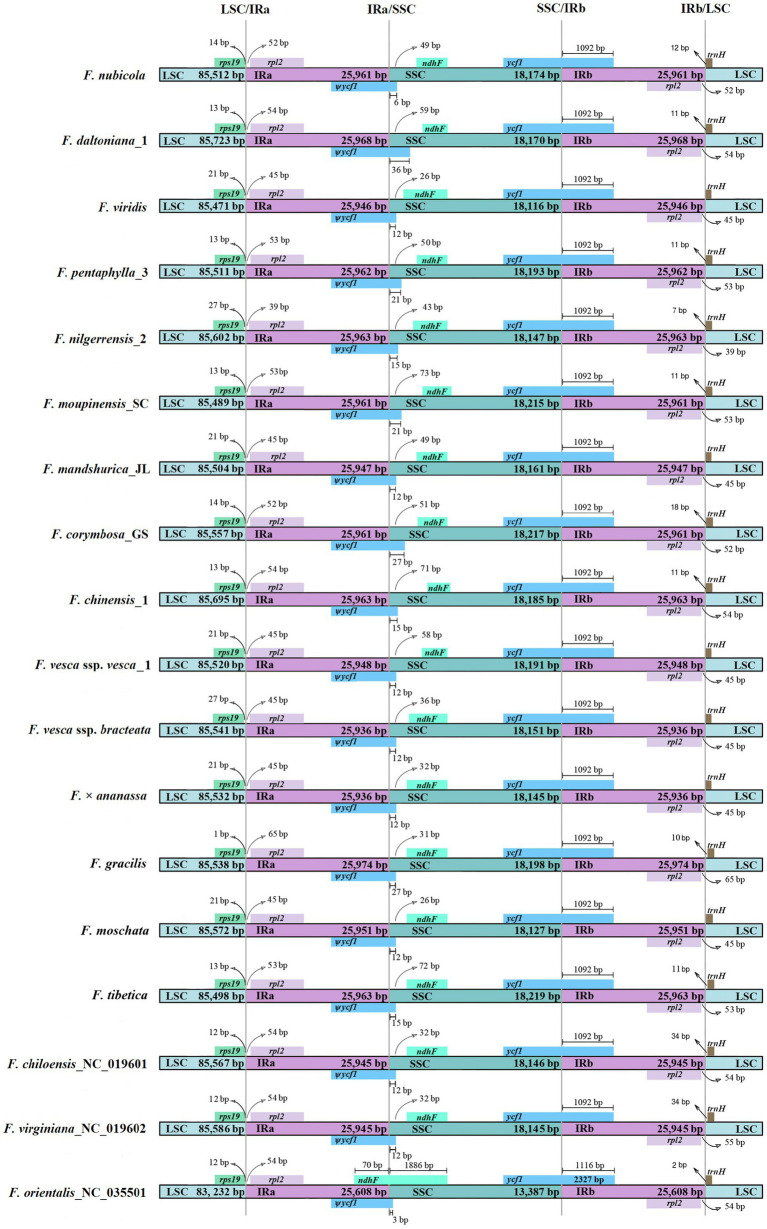
Comparison of the LSC, SSC, and IR border regions among 18 *Fragaria* chloroplast genomes. *Ѱ* is used to indicate pseudogenes.

The detailed comparisons of LSC, SSC, and IR boundaries in 19 *Fragaria* complete cp genomes are shown in Figure 2, revealing differences at boundary regions. There were five genes around borders of these regions, including *rps19*, *rpl2*, *ycf1*, *ndhF*, and *trnH*, in which *rpl2* and *ycf1* had two copies. Overall, *F. moschata*, *F. mandshurica_*JL, *F. viridis*, *F. vesca* ssp. *vesca*_1, *F. vesca* ssp. *bracteata*, and *F. × ananassa* showed higher structural similarity, with *rps19*, *rpl2*, and *trnH* genes found at the same distances from the boundaries. The *ycf1* gene in *Fragaria* species spanned 1,092bp from the SSC to IRb region, resulting in a non-functional *ѱycf1* fragment gene with the same length in IRa. Additionally, *ѱycf1* extended to the SSC region, with different distances ranging from 3bp (*F. orientalis*) to 37bp (*F. daltoniana*_1), with the cp genomes of *F. mandshurica_*JL, *F. viridis*, *F. vesca* ssp. *vesca*_1, *F. vesca* ssp. *bracteata*, *F. × ananassa*, *F. chiloensis*, and *F. virginiana* all showing the same gap of 13bp. Moreover, *ѱycf1* and *ndhF* had more length polymorphisms at the IRa/SSC border (Figure 2).

**Figure 3:**
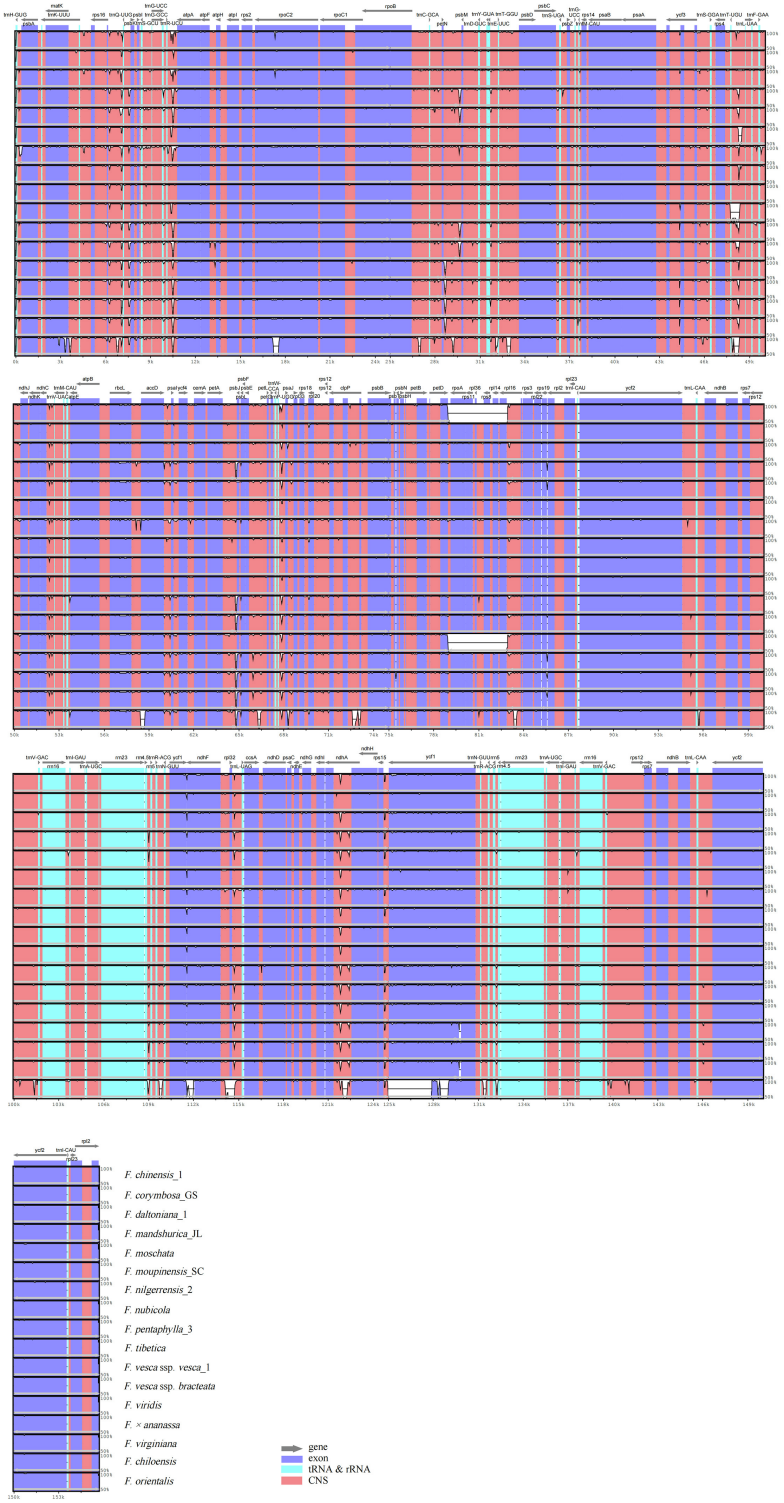
Visualized alignment of the *Fragaria* chloroplast genome sequences with annotations, using mVISTA. Each horizontal row shows the graph for the pairwise sequence identity with the *Fragaria gracilis* chloroplast genome sequence. The *x*-axis represents the base sequence of the alignment, and the *y*-axis represents the pairwise percent identity ranging from 50 to 100%. Gray arrows indicate the position and direction of each gene. Red indicates non-coding sequences (CNS); blue indicates the exons of protein-coding genes (exons); lime green indicates ribosomal RNA (rRNA) or transfer RNA (tRNA) sequences.

Annotation of the *F. gracilis* cp genome was used for plotting the total sequence identity of the 18 *Fragaria* cp genomes in mVISTA (Figure 3). Overall, there was a high similarity among *Fragaria* species, except *F. chinensis*_1, *F. viridis*, and *F. orientalis*. Furthermore, these results showed non-coding regions are more divergent than coding regions. In our research, the most divergent coding regions in the *Fragaria* cp genomes analyzed were *rpoC2*, *psbJ*, *ycf1*, and *ndhA*. In addition, the representative most divergent non-coding regions were *rps16*-*trnQ*, *trnR*-*atpA*, *petN*-*psbM*, *trnT*-*trnL*, *ndhC*-*trnV*, *petA*-*psbJ*, *trnP*-*psaJ*, *rpl32*-*trnL*, and *rps15*-*ycf1.*

**Figure 4:**
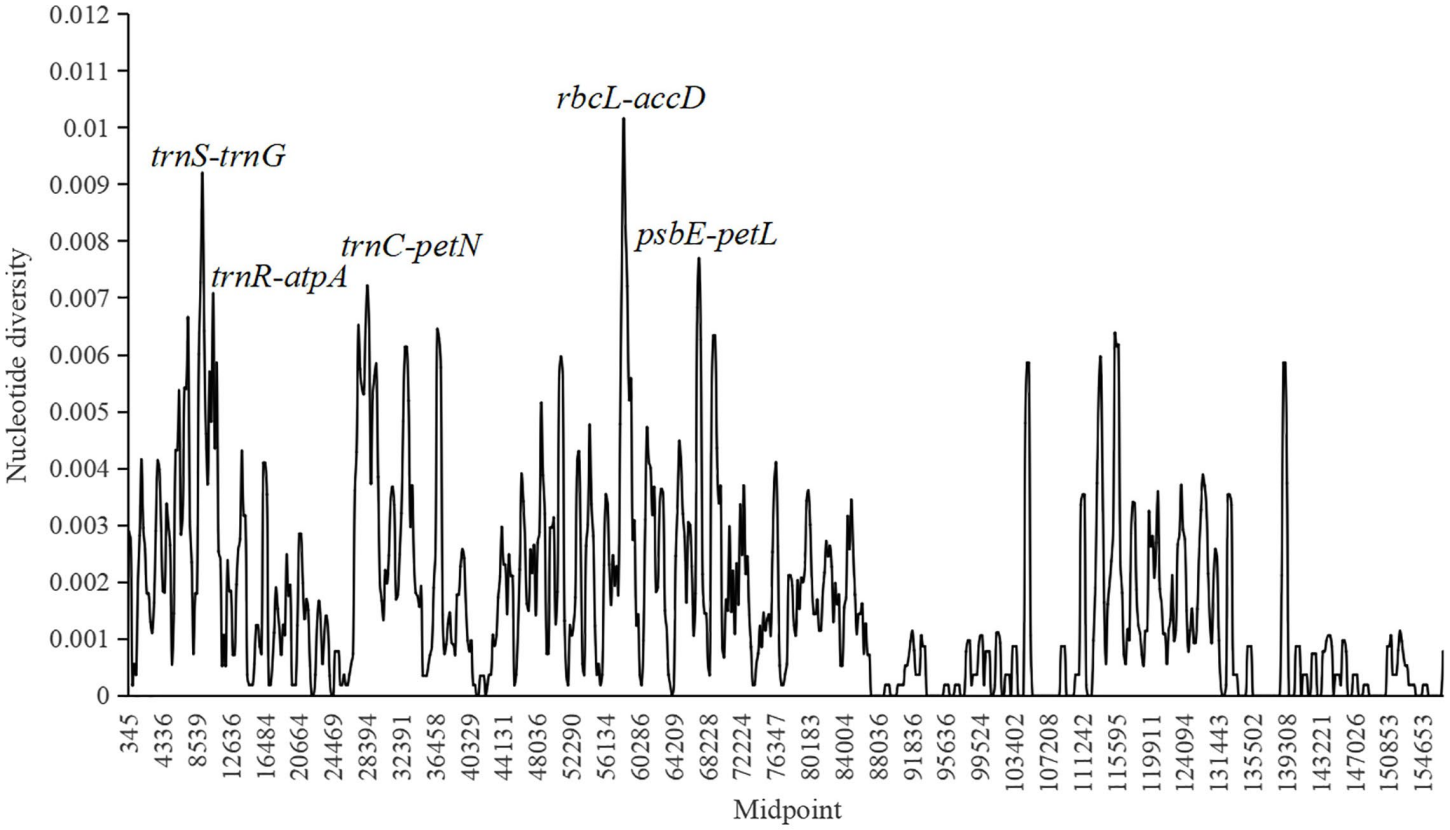
Sliding window analysis of 18 complete chloroplast (cp) genomes of *Fragaria* species (window length, 600bp; step size, 200bp; *x*-axis, position of the midpoint of a window; *y*-axis, nucleotide diversity of each window). Each highly polymorphic region of the *Fragaria* cp genomes is annotated on the graph.

Nucleotide diversity (*π*) was calculated to evaluate the sequence variability level in *Fragaria* cp genomes using DnaSP 6.0 software (Figure 4). The values ranged from 0 to 0.01016, and the average value was 0.00167. Four more polymorphic regions (*π*>0.007) were identified, including *trnS*-*trnG*, *trnR*-*atpA*, *trnC*-*petN*, *rbcL*-*accD*, and *psbE*-*petL*, in which *rbcL*-*accD* had the highest *π* value, and all of these regions were located in the LSC region.

### Phylogenetic Analysis

**Figure 5:**
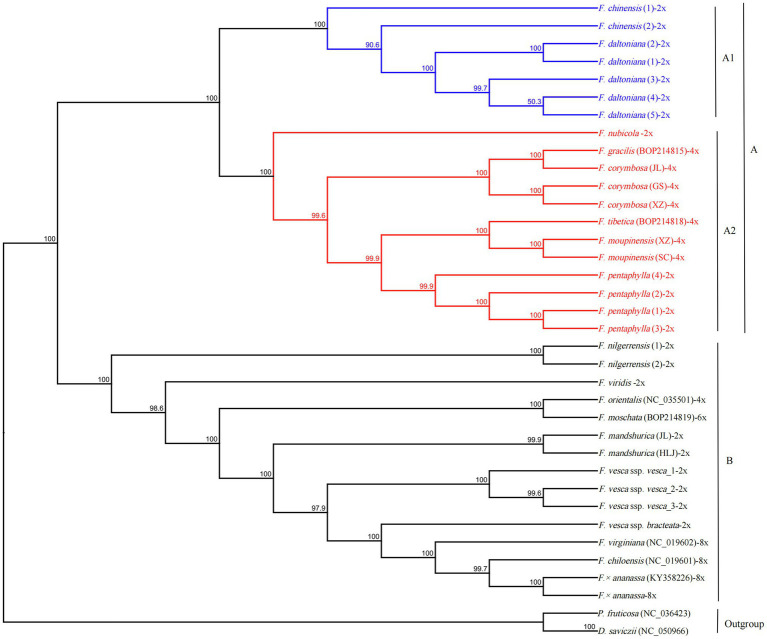
A maximum likelihood phylogenetic tree was reconstructed based on 34 *Fragaria* cp genomes. *Potentilla fruticosa* and *Drymocallis saviczii* were used as outgroups, and −2x, −4x, and −8x represent the different ploidies of *Fragaria* spp. Nodes marked with capital letters are discussed in the text.

The GTR+I+G model was selected as the best-fit evolutionary model by using Modeltest 3.7. The phylogenetic tree was constructed using 34 complete cp sequences of *Fragaria* species, with *P. fruticosa* and *D. saviczii* as outgroups. As shown in Figure 5, *Fragaria* species can be clustered into two groups, A and B, with 100% bootstrap support. Group A included two subgroups, A1 (*F. chinensis* and *F. daltoniana*) and A2. Subgroup A2 contained six *Fragaria* species (*F. nubicola*, *F. pentaphylla*, *F. corymbosa*, *F. moupinensis*, *F. gracilis* and *F. tibetica*) that are mainly distributed in western China. Group B was composed of the remaining species, which included *F. nilgerrensis*, *F. mandshurica*, *F. viridis*, *F. orientalis*, *F. moschata*, *F. × ananassa*, *F. chiloensis*, *F. virginiana*, *F. vesca* ssp. *vesca* and *F. vesca* ssp. *bracteata*. These latter species are from Europe and America, apart from *F. nilgerrensis*, which is mainly distributed in southeast Asia, *F. mandshurica*, which is distributed in northeast China, and *F. orientalis*, which is mainly found in northern Asia.

## Discussion

### Variations and Evolution of Whole Cp Genomes of *Fragaria* spp.

In this study, 27 cp genomes of *Fragaria* species were sequenced and found to range in size from 155,479 to 155,832bp, which falls within the cp genome size range for angiosperms but tends to be smaller than the cp genomes of other Rosaceae species (Palmer, 1985; Cheng et al., 2017). LSC regions showed the most difference in size, ranging from 85,471 to 85,726bp. Additionally, the inferred structures and gene contents were in accordance with previous research (Sun et al., 2021).

Overall, *Fragaria* cp genomes were highly conservative, both in sequence and structure. Analysis with mVISTA showed that there is high similarity among *Fragaria* species apart from *F. chinensis*_1, *F. viridis*, and *F. orientalis*. We also observed that most variable regions were located in LSC, and non-coding regions were more variable than coding regions. This is a common phenomenon in the cp genomes of most angiosperms (Nazareno et al., 2015; Cheng et al., 2017; Asaf et al., 2018; Tyagi et al., 2020). Additionally, some of the most divergent regions of *ycf1*, *rps16*-*trnQ*, *petN*-*psbM*, and *rpl32*-*trnL*, as shown in Figure 5, were consistent with previous research (Cheng et al., 2017), indicating that these regions indeed evolve rapidly in *Fragaria*.

The size differences among cp genomes in angiosperms may be caused by both the contraction and expansion of IR regions (Raubeson et al., 2007; Zhao et al., 2015, 2018). To elucidate this mechanism in *Fragaria*, we compared IR/SC boundaries of *Fragaria* cp genomes. In general, the distribution of border genes is conserved, but the distances between genes and the borders do differ somewhat. The distances between genes and IR/SC borders of *F. virginiana*, *F. orientalis*, and *F. × ananassa* are in accordance with the prior report by Cheng et al. (2017). *Fragaria mandshurica*, *F. viridis*, *F. moschata*, *F. × ananassa*, *F. vesca* ssp. *vesca* and *F. vesca* ssp. *bracteata* showed the same gap between *rps19*, *rpl2*, *ycf1*, *trnH*, and IR/SC junctions, which may explain why these cp genomes are more conserved. Additionally, some species also exhibited the same distances between the *rps19* and LSC/IRa border, including *F. pentaphylla*, *F. moupinensis*, and *F. tibetica.* These results also indicated a low level of molecular divergence in the genus *Fragaria*.

Additionally, *rpl2* and *rps19* differed in their distances from the LSC/IRa border, which may be owing to IR contraction and expansion. Compared with *F. × ananassa*, the IR regions of *F. pentaphylla*, *F. daltoniana*, *F. nubicola*, *F. chinensis*, *F. corymbosa*, *F. moupinensis*, *F. orientalis*, *F. gracilis*, *F. tibetica*, *F. virginiana*, and *F. chiloensis* expanded to different degrees. Therefore, IR regions of these species are longer than that of *F. × ananassa*. For the other species in the family Rosaceae, such as *Malus* (Terakami et al., 2012), *Prinsepia* (Wang et al., 2013), *Pyrus* (Li et al., 2018), and *Prunus* (Kim et al., 2019), *rps19* crossed the LSC region and IR region. Additionally, *rps19* extended to the IRa region, resulting in the presence of *ѱrps19* having the same length within IRb region. Notably, we found that *rps19* was located inside the LSC region. The contraction of *rps19* inside the LSC region would result in the size of the *Fragaria* cp genomes being smaller than that of other species in Rosaceae.

### Phylogenetic Analysis

The similar morphology of most *Fragaria* spp. and the geographical overlap in ranges may lead to taxonomic confusion among collected specimens (Johnson et al., 2014) and finally result in misjudgment of phylogenetic relationship. To explore the evolutionary relationships among *Fragaria* species, we constructed a phylogenetic tree of *Fragaria* species based on whole cp genomes with more than 99% bootstrap support across all nodes. Our results showed high consistency with previous results, especially for species clustering in group B (Njuguna et al., 2013; Sun et al., 2021). Additionally, our results include multiple individuals of each species from different collection sites, which increases the reliability of the phylogenetic analysis.

The phylogenetic analysis showed that *F. × ananassa* and two octoploids, *F. chiloensis* and *F. virginiana*, formed a group, which was in accordance with the inferred origin of *F. × ananassa* from a hybridization between *F. virginiana* and *F. chiloensis* (Staudt, 1962, 1989). In addition, the two octoploids were considered to share a common maternal ancestor that may be *F. vesca* or *F. mandshurica* (Harrison et al., 1997; Rousseau-Gueutina et al., 2009; Dimeglio et al., 2014). Fortunately, we observed that *F. vesca* ssp. *bracteata* is evolutionarily closely related to the two octoploids. Similar results were also presented by Sun et al. (2021). Additionally, Njuguna et al. (2013) and Edger et al. (2019) suggested that *F. vesca* ssp. *bracteata* was probably the last diploid progenitor to the octoploid species. Thus, the conclusion that *F. vesca* ssp. *bracteata* is an ancestor of octoploid species was strengthened. However, the other ancestors of the octoploid species remain uncertain.

The evolutionary relationships of *F. nubicola*, *F. pentaphylla*, *F. chinensis*, *F. daltoniana*, *F. corymbosa*, *F. moupinensis*, *F. gracilis*, and *F. tibetica* have never been clear (Rousseau-Gueutina et al., 2009; Sun et al., 2021). These species are mainly distributed in Western China (Lei et al., 2017) and, in our results, they were clustered into group A. In group A2, diploid *F. pentaphylla* was sister to the tetraploids *F. moupinensis* and *F. tibetica* with 99.9% bootstrap support (Figure 5), which was consistent with previous research (Njuguna et al., 2013; Sun et al., 2021). In addition, *F. pentaphylla* had been suggested to be the diploid ancestor to *F. moupinensis* (Rousseau-Gueutina et al., 2009; Kamneva et al., 2017). Lei et al. (2017) hypothesized that *F. tibetica* is a descendant of *F. pentaphylla* based on their runner branching and number of leaflets. Thus, it can be hypothesized that the tetraploid species *F. moupinensis* and *F. tibetica* may share the same female parent of *F. pentaphylla*, which is supported by their more similar morphological characteristics (Lei et al., 2017) and overlapping distribution in Southwestern China (Staudt, 1989; Johnson et al., 2014; Lei et al., 2017).

In our study, we revealed a sister relationship between *F. corymbosa* and *F. gracilis*, which was consistent with Rousseau-Gueutina et al. (2009). And *F. corymbosa* and *F. gracilis* have some similar morphological characteristics, such as runners are filiform and monopodial, petioles and peduncles have spreading hairs, fruits are red and tasteless, calyx is reflexed, etc. (Lei et al., 2017). So our results strengthened the point that *F. corymbosa* and *F. gracilis* may have the same ancestor (Rousseau-Gueutina et al., 2009). In addition, *F. corymbosa* and *F. gracilis* may be the descendent of *F. chinensis* (Staudt, 2009; Yang and Davis, 2017). Notably, in this study, all accessions of *F. chinensis* and *F. daltoniana* were clustered into group A1, in contrast with the findings of previous studies (Yang and Davis, 2017; Sun et al., 2021). Therefore, future research should explore the relationship among *F. chinensis*, *F. corymbosa*, and *F. daltoniana*. In the future, multiple molecular markers, including cp and nuclear sequence data from more samples from different geographical populations, should be combined with geographical distribution data to analyse ancestral state reconstruction to clarify their phylogenetic ancestor relationships among *F. moupinensis* and *F. tibetica*; *F. chinensis*, *F. corymbosa*, and *F. daltoniana*; *F. vesca* ssp. *bracteata* and the other octoploid species.

Chloroplast capture is an important process of plant evolution (Okuyama et al., 2005). Hybridization and repeated backcross, the cytoplasm of one species is replaced by the cytoplasm of another species through gene flow infiltration, so that the genetic components of the species not only have nuclear genome components inherited from parents, but also capture new chloroplast gene components (Fehrer et al., 2007). More and more studies have proved the phenomenon of organelle DNA introgression (Du et al., 2011), and the phenomenon of chloroplast introgression between plant species has also been observed in previous studies on hazelnut (Hu et al., 2020). In this study, the phylogeographical relationships among *Fragaria* species were not declared for the lack of geographical population collections. However, clear geographical patterns (Western China, Southwestern China, and Northeastern China) have been clearly inferred in phylogenic tree figure. Chloroplast capture could be another explanation why the chloroplast genome analysis does not appear to reflect the species phylogeographical relationship (Tsitrone et al., 2003). Further research should be conducted by combining multiple molecular tools (e.g., nuclear DNA sequences) together with more comprehensive sampling to clarify if chloroplast capture does occur in *Fragaria* genus.

### Candidate Barcoding Sequences for *Fragaria*

Species identification based on morphology is affected by season, environment, and human factors, which may cause results to be unreliable (Yang et al., 2020). In recent years, DNA barcoding has been widely used to promote accurate species identification owing to its clear advantages (Tegally et al., 2019; Phi et al., 2020; Islam et al., 2021). The ideal DNA barcode would be a single locus that could be universally amplified and sequenced across a broad range of taxa and provide sufficient variation to reliably distinguish among closely related species (Song et al., 2017). Many introns, coding regions, and intergenic regions, such as *trnL*-*trnF* (Potter et al., 2000), *accD*-*psaI* (Wang et al., 2017), *ycf1*-*ndhF* (Amar, 2020), *matK*, and *trnK* (Hilu et al., 2008), have been used as barcodes for constructing phylogenetic relationships. In this study, with the threshold of nucleotide diversity as 0.007, the five intergenic regions, *trnS*-*trnG*, *trnR*-*atpA*, *trnC*-*petN*, *rbcL*-*accD*, and *psbE*-*petL* were found to be the most divergent and provide potential information for species identification and phylogenetic analyses of *Fragaria*. Among them, three intergenic regions, including *trnS*-*atpA*, *rbcL*-*accD*, and *psbE*-*petL* were also suggested in Sun et al. (2021). However, the threshold of nucleotide diversity used in Sun et al. (2021) was only 0.006, resulting in a low nucleotide diversity of the selected candidate barcoding sequences. In addition, the amplified fragments of the selected intergenic regions *trnS-atpA* more than 2,000bp in length would reduce the success of the sequencing. In our study, *trnS-trnG* and *trnR-atpA* are located in *trnS-atpA* regions and the length of these two regions are about 700bp, which will result in the high success of sequencing. Therefore, *trnS-trnG* and *trnR-atpA* are more suitable than *trnS-atpA* to be the potential candidate barcoding sequence.

## Conclusion

This study provides 27 complete cp genome sequences of 11 wild *Fragaria* species. Comparative analysis of cp genomes of *Fragaria* species revealed that their genome structure is highly conserved. However, IR expansion or contraction was observed among different *Fragaria* cp genomes, resulting in cp genomes of different sizes. Five identified highly variable gene regions (*trnS*-*trnG*, *trnR*-*atpA*, *trnC*-*petN*, *rbcL*-*accD*, and *psbE*-*petL*) showed strong potential for species identification and phylogenetic relationship construction in the genus *Fragaria*. Phylogenetic analysis indicated that *F. vesca* ssp. *bracteata* may be one of the progenitor species of octoploids. Similarly, we hypothesize that *F. pentaphylla* is one of the progenitors of *F. corymbosa* and *F. tibetica*. The analysis of multiple molecular markers combined with morphological characters would be helpful for future research to test this hypothesis.

## Data Availability Statement

The datasets presented in this study can be found in online repositories. The names of the repository and accession number(s) can be found below: NCBI repository, accession numbers MZ851747 and MZ851773.

## Author Contributions

JL designed the research. CL, CC, YT, ZS, and MJ performed the research and analyzed the data. CL wrote the first draft of the manuscript. All authors commented on previous versions of the manuscript. All authors contributed to the article and approved the submitted version.

## Funding

This work was financially supported by the Ten Thousand Talent Program of Zhejiang Province (No. 2019R52043) and the National Natural Science Foundation of China (No. 31261120580).

## Conflict of Interest

The authors declare that the research was conducted in the absence of any commercial or financial relationships that could be construed as a potential conflict of interest.

## Publisher’s Note

All claims expressed in this article are solely those of the authors and do not necessarily represent those of their affiliated organizations, or those of the publisher, the editors and the reviewers. Any product that may be evaluated in this article, or claim that may be made by its manufacturer, is not guaranteed or endorsed by the publisher.

## Footnotes

^1^ https://chlorobox.mpimp-golm.mpg.de/geseq.html
